# Dose–response relationship of treadmill perturbation-based balance training for improving reactive balance in older adults at risk of falling: results of the FEATURE randomized controlled pilot trial

**DOI:** 10.1186/s11556-025-00375-w

**Published:** 2025-05-16

**Authors:** Natalie Hezel, Theresa Buchner, Clemens Becker, Jürgen M. Bauer, Lizeth H. Sloot, Simon Steib, Christian Werner

**Affiliations:** 1 Geriatric Center, Medical Faculty Heidelberg, Heidelberg University, Heidelberg, Germany; 2 Optimization, Robotics, and Biomechanics, Institute of Computer Engineering, Heidelberg University, Heidelberg, Germany; 3 Translational and Clinical Research Institute, Newcastle University, Newcastle, United Kingdom; 4 Department of Human Movement, Training and Active Aging, Institute of Sports and Sports Sciences, Heidelberg University, Heidelberg, Germany; 5 Network Aging Research Heidelberg, Heidelberg University, Heidelberg, Germany

**Keywords:** Exercise, Falls, Postural control, Dose–response relationship, Frail older adults, Patient acceptance of health care, Feasibility studies

## Abstract

**Background:**

The inability to appropriately react to balance perturbations is a common cause of falls. Perturbation-based balance training (PBT) is especially beneficial for improving reactive balance and shows high potential for fall prevention. However, its dose–response relationship, feasibility, and acceptability remain to be determined among older adults at risk of falling. The FEATURE study aimed to compare the efficacy of two treadmill PBT protocols with different session numbers to improve reactive balance, and to evaluate their feasibility and acceptability in this population.

**Methods:**

In this randomized controlled pilot trial, 36 older adults at risk of falling were allocated to receive either six (6PBT) or two treadmill PBT sessions (2PBT). Reactive balance in standing (Stepping Threshold Test [STT]) and walking (Dynamic Stepping Threshold Test [DSTT]) was assessed as primary outcome at baseline (T1), post-intervention (T2), and 6-week follow-up (T3). Secondary outcomes included measures on physical, psychological, and cognitive functioning. Feasibility was assessed via PBT adherence, planned perturbations completed, and adverse events; acceptability via questionnaire. Between-group changes over time were compared using repeated-measures analyses of variance with Bonferroni-corrected post-hoc tests. Data analyses followed the intention-to-treat principle.

**Results:**

A significant time effect was observed for the DSTT (*p* = 0.008), with both groups significantly improving from T1 to T2 (*p*s < 0.01). A significant interaction effect (*p* = 0.027) revealed that only the 6PBT group maintained these improvements (T1 vs. T3: *p* < 0.001) and scored significantly higher than the 2PBT group at T3 (*p* = 0.015). No significant interaction effects were found for the STT or any secondary outcome, but improvements over time were observed for dynamic balance, gait capacity, functional mobility, physical activity, concerns about falling, and executive functioning (time effects: *p*s < 0.05). PBT adherence, planned perturbations completed, and acceptability were high in both groups, with no significant between-group differences. No intervention-related serious adverse events were reported.

**Conclusions:**

Findings suggest that a low number of treadmill PBT sessions can lead to task-specific improvements in reactive balance during walking, with a higher practice dose enhancing sustainability. Treadmill PBT appears feasible and well-accepted among older adults at risk of falling, regardless of sessions received.

**Trial registration:**

DRKS00030805; prospectively registered December 14, 2022.

**Supplementary Information:**

The online version contains supplementary material available at 10.1186/s11556-025-00375-w.

## Background

About one third of older adults experience a fall each year [1], with the likelihood of falls increasing with age. Globally, falls are the second leading cause of unintentional injury-related deaths [2], often resulting in devastating consequences for both individuals and their social environment. As the ageing population grows due to demographic shifts, the incidence and impact of falls are anticipated to rise [3]. Consequently, fall prevention has become a major public health issue, underscoring the urgent need for effective strategies.

Slipping and tripping are the most common circumstances in which falls and fall-related injuries occur among older adults [4, 5]. Reactive balance, defined as the ability to recover from an unexpected threat to balance, such as a slip or trip [6], may therefore play a crucial role in preventing falls in daily life. While evidence-based fall prevention exercises include activities to train static and dynamic balance, muscle strength and functional capacity, they often lack the task specificity to target the ability to effectively recover stability and avoid falling after sudden and unexpected balance disturbances [7]. In contrast, perturbation-based balance training (PBT) applies repeated, externally induced mechanical perturbations to elicit rapid reactions for regaining postural stability in a safe and controlled environment [8]. This task-specific training is considered the optimal exercise for improving reactive balance [9] and shows high potential as an efficient fall prevention strategy for older adults, although the current body of evidence remains limited. Some studies have demonstrated an impressive reduction in fall rates by about 50% over 6 to 12 months after one to eight PBT sessions (30–60 min per session) [10–12]. This corresponds to approximately twice the effect with a significantly lower training volume compared to other evidence-based fall prevention programs, for which a fall reduction of 23% and a training duration of 2 to 3 h per week for at least 12 months have been recommended [13]. However, there are also other studies in older adults that could not document such fall-reducing effects of PBT with similar low training volume (1–4 PBT sessions, 20–30 min per session) [14, 15], indicating that further research is needed to evaluate the effectiveness of PBT for fall prevention.

Previous studies in older adults varied not only in the number of PBT sessions but also in the number of applied perturbations per session, ranging from 20 up to 120 trials [10, 11, 14–18], or individualized amounts [19, 20]. Additionally, several different methods were used for applying PBT, such as overground walkways with pop-up obstacles, low-friction movable platforms, and/or slippery surfaces (e.g., oil layer) [10, 16–18] or specialized treadmills allowing for sudden belt accelerations/decelerations [11, 14, 15], and lateral platform displacements [19, 20]. Further differences include perturbation types (anterior [12], posterior [10, 15, 17], anterior–posterior [AP] [11, 14, 16, 18], AP and mediolateral [ML] perturbations [19, 20]) and intensity used in these studies (e.g., based on participant rating, trainer judgement, and/or a combination of both [11, 14, 19, 20], or predetermined by technical specifications of the PBT system [10, 16, 18]).

While most of these studies reported improvements in participants'reactive balance and/or reduced fall rates following exposure to various perturbation paradigms [10–12, 14, 16, 17, 19], the heterogeneity in perturbation dose, type, and intensity limits the understanding of the specific mechanisms driving these effects and how adaptations are retained long-term.

Studies providing insight into dose–response relationship of PBT suggest a non-linear pattern in healthy older adults at low risk of falling. Rapid initial improvements in reactive balance (“first-trial effect”) have been observed in trial-to-trial adaptations to a small number of repeated overground gait-slip perturbations, followed by a subsequent decay and plateauing of gains as practice dosage increases [21, 22]. In addition, increasing the practice dosage of treadmill PBT (24 vs. 40 perturbations) has been shown to provide no additional immediate generalization effects on reactive balance during perturbed overground walking [17]. In contrast, among more impaired older adults with lower neuromotor control and sensory system capabilities, adaptions of reactive balance have been reported to occur at a slower rate, suggesting that a greater total number of perturbation trials over a given exercise period may be required to achieve significant improvements [23]. Nevertheless, there remains a critical need for research into the dose–response relationship of PBT in older adults, particularly those at risk of falling [8, 23].

In general, previous PBT studies have so far often focused on healthy older adults with low fall risk [10, 11, 14–18] or patients with specific chronic conditions (e.g., Parkinson’s disease [24], chronic obstructive pulmonary disease [25]). Only a few studies have shown PBT is feasible [20] and effective for improving reactive balance in older adults at risk of falling [26–28]. Further research is necessary to better understand and optimize PBT for this vulnerable population [8].

Despite the challenging nature of PBT in mimicking near fall situations [8] and higher potential for adverse events, such as anxiety or pain compared to other exercise interventions [29], there is also limited knowledge about its acceptability. Only two qualitative studies [30, 31] and one quantitative study [32] have shown PBT to be perceived as acceptable among older adults. However, treadmill PBT protocols with different practice dosages have not yet been compared for acceptability in older adults at risk of falling. Ensuring the acceptability of training interventions is essential for successful implementation, as even the most effective approaches can fail if not embraced by the target group.

The primary aim of this study was to gain insights into the dose–response relationship of PBT by comparing the efficacy of a 6-session (6PBT) versus a 2-session PBT (2PBT) delivered on a treadmill for improving reactive balance in older adults at risk of falling. We hypothesized that 6PBT yields significantly greater improvements in reactive balance compared to 2PBT. The secondary aim was to evaluate the feasibility and acceptability of the treadmill PBT protocols.

## Methods

### Study design

The FEATURE study was a monocentric, assessor-blinded, randomized controlled pilot trial with a 6-week intervention period and a 6-week follow-up period (T1 = baseline, T2 = post-intervention, T3 = follow-up), conducted between January and November 2023 in Heidelberg, Germany. Details on the study protocol were reported previously [33]. There were no significant deviations from the protocol. Reporting in this article followed the CONSORT (Consolidated Standards of Reporting Trials) reporting guidelines for parallel group randomized trials.

### Participants

Participants were recruited between January 2023 and July 2023 from a senior fitness club (Rehabilitation Sports in Geriatrics [REGE] e.V.), associated with a German geriatric hospital (Agaplesion Bethanien Hospital Heidelberg). Members of the REGE e.V. regularly attend one 90-min training session per week, focusing on strength and balance exercises. Inclusion criteria were age ≥ 65 years, risk of falling (Timed Up and Go [TUG] > 12 s [34], habitual gait speed < 1.0 m/s [35], and/or fall(s) in past 12 months [3]), and able to walk ≥ 2 min without walking aid. Exclusion criteria were cognitive impairment (Mini-Mental State Examination [MMSE] ≤ 24 pt.) [36] and severe neurological, cardiovascular, metabolic, or psychiatric disorders.

### Randomization and blinding

Participants were randomized after baseline assessment into one of the two intervention arms through block-randomization with a 1:1 allocation ratio stratified by treadmill experience (Do you exercise regularly on the treadmill during your REGE training session? [yes vs. no]) and habitual gait speed (≥ 1.0 m/s vs. < 1.0 m/s). The study coordinator (N.H.) carried out randomization. If participants withdrew from the intervention, they remained eligible for post-intervention and follow-up assessments. Assessors were blinded to the group allocation.

### Interventions

Both intervention arms are described in detail, along with a TIDieR (Template for Intervention Description and Replication) checklist, in the study protocol [33]. Intervention sessions were embedded in the participants’ once-weekly, 90-min REGE e.V. training session and lasted for about 30 min. The intervention period was 6 weeks, with 1 training session per week. Sports science students were trained to deliver the training protocol to ensure standardization across different trainers. The 6PBT group received six PBT sessions (week 1–6), while the 2PBT group performed two PBT sessions (week 1 + 6) plus four conventional treadmill training (CTT) sessions without perturbations (weeks 2–5).

All PBT sessions were conducted on the BalanceTutor™ (MediTouch, Netanya, Israel). Participants were secured by an overhead safety harness system. Each PBT session consisted of 40 unannounced perturbations in total, divided into five blocks of 1.5 to 3.5 min, with 8 perturbations each. Participants experienced AP perturbations in block 1 and 2, ML perturbations in block 3 and 4, and AP and ML perturbations in block 5. Directions of the perturbations were randomized in each block, as was the time interval between perturbations ranging from 10 to 25 s. Perturbations were induced in each block to an equal number at the swing phase of the left and right leg, respectively, determined by the automatic detection of the specific gait swing phase for perturbation timing of the BalanceTutor™. Comfortable treadmill speed was determined at the first PBT session [14, 33] and used in all subsequent PBT sessions. The BalanceTutor™ allows for 30 different levels of perturbation magnitudes in each direction. Perturbation magnitude was individually progressed based on combined ratings from two 5-point Likert scales for self-perceived difficulty (1 = easy, 5 = too hard) and anxiety (1 = not at all, 5 = extremely) [16, 37], assessed with participants after each training block, targeting an average score of 2 to 3 in blocks 1 and 3, and 3 to 4 in blocks 2, 4, and 5. Perturbation magnitudes were increased or decreased if combined ratings fell below or above the targeted range.

The CTT sessions were conducted on a different medical treadmill (pluto med,h/p/cosmos sports & medical gmbh, Nussdorf-Traunstein, Germany), which is regularly used in the REGE e.V. Each CTT session also consisted of five 3-min blocks at the comfortable treadmill speed determined in the first PBT session, but without applying perturbations. The walking duration of the CTT sessions was similar to that of the PBT sessions.

### Primary outcomes

#### Stepping Threshold Test (STT)

The STT assesses static reactive balance on a perturbation treadmill that provides unannounced AP and ML surface translation perturbations of increasing magnitudes in random order [38, 39]. Perturbations gradually increases over six levels, and participants, secured by a harness system, are instructed to use as few compensatory steps as possible. Single-step and multiple-step thresholds, defined as the level (1 to 6) at which a participant requires one step or multiple steps (≥ 2) to regain balance, for each perturbation direction are determined, and summed to yield a STT total score (8 to 56 points). If the STT is terminated early due to a fall or excessive fear of the participant, thresholds are set one level above the last completed level. Stepping behavior can be assessed using an all-step count evaluation (ACE) and a direction-sensitive evaluation (DSE) [38, 39]. The STT was performed on the BalanceTutor™ and video recorded by two cameras (HERO9 Black, GoPro, San Mateo, CA, USA) positioned at about 35° fronto-lateral to the participant and recording at a frame rate of 60 Hz. Stepping behavior was evaluated from the video recordings by one rater (N.H.) to prevent inter-rater variability. Convergent validity of the STT has been documented via associations with various mobility, psychological, and cognitive measures in fall-prone older adults [38, 39], as well as its discriminant validity in distinguishing older adults fallers and non-fallers [39]. In addition, the video-based evaluation strategy of the STT has been shown to be inter-observer reliable in healthy adults and stroke patients (Kappa coefficient = 0.89–0.99) [40].

#### Dynamic Stepping Threshold Test (DSTT)

The DSTT, a modified version of the STT, was used to assess dynamic reactive balance [33]. Participants walked on the BalanceTutor™, also secured by the harness system, with 70% of their habitual overground walking speed and received unannounced perturbations. The DSTT protocol included five levels with increasing perturbation magnitudes gradually increasing in steps of 5 (level 1: magnitude = 5, level 5: magnitude = 25). Each level contained eight different perturbations (4 directions [left, right, forward, backward] × 2 swing phases [left/right leg]) performed once per level in random order and at random intervals of 10 to 19.5 s. Perturbation timing at the specific swing phase was determined by the automatic detection of the BalanceTutor™. Participants were instructed to counteract the perturbations and return to normal walking as quickly as possible. The DSTT was stopped in case of a fall in the harness system or if excessive anxiety was reported. For each of the five levels, a subscore was calculated as follows: level number × number of successfully completed perturbations (e.g., level 3 × 4 perturbations = 12 points). Each level subscore was summed to yield a DSTT total score ranging from 0 to 120 points. More detailed information on the DSTT protocol was provided in the study protocol [33].

Both the STT and DSTT were performed on the BalanceTutor™ with participants facing in the opposite direction to that used during the PBT.

### Secondary outcomes

Global balance was assessed with Brief Balance Evaluation Systems Test (Brief-BESTest) [41], and dynamic balance with the Four-Square Step Test (FSST) [42].

Gait capacity was measured through spatio-temporal gait parameters (gait speed, cadence, step time, stride length, total double support, walk ratio) captured via the Mobility Lab (APDM Inc., Portland, OR, USA) during a 10-m walking test at habitual pace [43], and a 2-min walk test (2MWT) on a 10-m course [44].

Functional mobility was evaluated using the TUG [45] and SPPB [46].

Physical activity (PA) was recorded using an activity sensor (GT9X Link or wGT3X-BT; ActiGraph LLC, Pensacola, FL, USA) worn on the wrist of the non-dominant hand for five consecutive days during awake time, except for water-related activities (e.g., bathing, showering, swimming). Data were processed in 60-s epochs using the ActiLife software (version 6.13.4, ActiGraph LLC, Pensacola, FL, USA). A wear time of ≥ 600 min per day was considered a valid day, and only ≥ 3 valid days (including 2 weekdays and 1 weekend day) were used as the criterion to include data into the analysis. PA outcomes included mean daily energy expenditure (metabolic equivalent of tasks, METs), mean daily duration (min) in moderate-to-vigorous PA (MVPA ≥ 2690 cpm), mean daily step count, and maximum step count per walking bout.

Concerns about falling was assessed using the Short Falls Efficacy Scale-International (Short FES-I) [47].

Executive functioning was tested using the Trail Making Test (TMT, parts A and B) [48].

Maximum perturbation magnitude for AP and MP directions completed was documented for the first and last PBT session by trainers.

Feasibility of the PBT was assessed via the adherence rate to the scheduled PBT sessions, dropout rate and reasons during the intervention, number of perturbations performed, proportion of planned perturbations completed, and adverse events during PBT sessions.

Acceptability of the PBT was evaluated post-intervention (T2) using a self-designed questionnaire based on the Theoretical Framework of Acceptability (TFA; lowest acceptability = 7 pt., highest acceptability = 35 pt.), covering one item for each of seven dimensions (affective attitude, burden, ethicality, intervention coherence, opportunity cost, perceived effectiveness, self-efficacy), rated on a 5-point Likert scale (1 = strongly disagree to 5 = strongly agree) [33, 49].

### Descriptive measures

Age, gender, education (years), body mass index, presence of chronic disease, fall history in the last 12 months, frailty status according to the Fried’s frailty criteria (unintentional weight loss, exhaustion, low PA, slowness, and weakness), global cognition (MMSE), depressive symptoms (5-item Geriatric Depression Scale), and subjective health status (EuroQol-5 dimension visual analogue scale) were assessed for participants’ characteristics.

### Sample size

The sample size was determined a priori based on recommendations for pilot randomized controlled trials (RCT) [50]. To detect a moderate effect size (0.5) for the differences between two groups with a statistical power (1 − *β*) of 0.90 and a two-sided significance level (*α*) of 0.05,

Fifteen participants per group were recommended. Accounting for an expected dropout rate of 15% [51, 52], the final sample size increased to 18 participants per group.

### Statistical analysis

Group differences (6PBT vs. 2PBT; dropouts vs. completers) were analyzed by *χ*^2^-tests or Fisher’s exact tests, Mann–Whitney *U* tests, or *t*-tests for independent samples. Two-way repeated-measures analyses of variance (ANOVA) were conducted with time (T1, T2, T3) as the within-subject factor and group (6PBT, 2PBT) as the between-subject factor to compare changes over time between groups. ANOVAs for primary outcomes were adjusted for treadmill experience and gait speed to account for stratification variables [53]. Post-hoc tests with Bonferroni correction for multiple comparisons were applied when significant interaction or time effects were observed, with corrected *p*-values reported (*p*_*Bonf*_). Extreme outliers in the outcomes (except for feasibility outcomes) were identified as values > 3 interquartile ranges (IQR) beyond the 25 th or 75 th percentiles and replaced with the nearest non-outlying value (winsorization) to reduce potential bias from disproportionate influence of extreme values while preserving overall data integrity in the small sample. Extreme outliers were identified only for the FSST (7 out of 89, 7.9%) and TUG (4 out of 92, 4.3%) but not for the primary outcomes. Mean daily step count, mean daily MVPA duration, and TMT-B were analyzed in ANOVA after natural log-transformation due to non-normally distributed residuals. All analyses were performed according to the intention-to-treat principle. Missing data were replaced by multiple imputation by chained equations with predictive mean matching as imputation method (20 imputations, 10 iterations), assuming data were missing at random. The imputation model included all outcome, descriptive, randomization, and stratification variables. Rubin’s rules were used to pool estimates (means, standard errors/deviations, *p*-values) across the multiple imputation datasets for parametric tests, while the median rule was used to pool estimates (medians, IQRs, *p*-values) for non-parametric tests. Complete-case analyses were also conducted as sensitivity analysis to investigate the robustness of the findings, with boxplots of the primary outcomes to illustrate the original data distribution (Additional file 1). Effect sizes were given as partial eta squared (*η*_*p*_^2^) and interpreted as small (*η*_*p*_^2^ < 0.06), moderate (0.06 ≥ *η*_*p*_^2^ < 0.14), or large effects (*η*_*p*_^2^ ≥ 0.14) [54]. Statistical significance was set at *p* < 0.05. All statistical analyses were performed using IBM SPSS version 29.0 (IBM Corp., Armonk, NY, USA).

## Results

### Participant characteristics

**Fig. 1:**
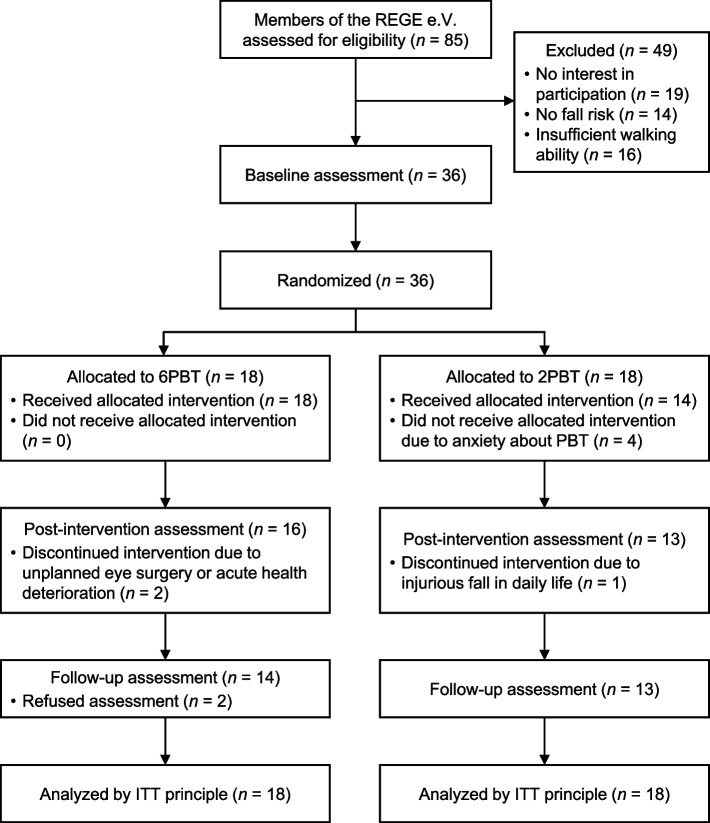
Flowchart for enrollment, allocation, intervention, assessment, and data analysis. REGE e.V. = Rehabilitation Sports in Geriatrics, 6PBT = six-session perturbation-based balance training, 2PBT = two-session perturbation-based balance training and four-session conventional treadmill training, ITT = intention-to-treat

Out of 85 screened REGE e.V. members, 36 met the inclusion criteria, provided written informed consent for participation, and were randomized into 6PBT (*n* = 18) and 2PBT (*n* = 18) (Fig. 1). The total sample included community-dwelling, cognitively intact (MMSE = 28.2 ± 1.5 pt.) older adults (age = 80.3 ± 5.4 years, females: *n* = 26, 72.2%) (Table 1). More than 80% of the participants (*n* = 30) reported having at least one chronic disease (e.g., arthrosis, hypertension, osteoporosis), one-third (*n* = 13, 36%) had a fall history, and more than half (*n* = 19, 53%) were categorized as pre-frail or frail. Physical capacity was mildly impaired, as indicated by a mean TUG duration of 12.2 ± 4.8 s, a mean SPPB score of 9.9 ± 2.5 pt., and a mean gait speed of 0.84 ± 0.16 m/s. Two-thirds reported at least moderate concerns about falling (Short FES-I > 9: *n* = 24, 66.7%). PA behavior was relatively high, with a median daily step count of 8435 [IQR 7742–8535] steps. No significant group differences were observed in any participant characteristics (*p* = 0.056–0.999) or primary and secondary outcomes at baseline (*p* = 0.071–0.968), indicating successful randomization (Table 1).

**Table 1:** Participant characteristics

Variable	Total (*n* = 36)	6PBT (*n* = 18)	2PBT (*n* = 18)	*p*
Age, years	80.3 ± 5.4	79.6 ± 4.9	80.9 ± 5.8	0.447
Female, *n*	26 (72.2)	13 (72.2)	13 (72.2)	> 0.999
Education, years	13.1 ± 3.4	14.2 ± 3.2	12.1 ± 3.3	0.056
BMI, kg/m^2^	25.9 ± 3.7	24.9 ± 3.1	26.9 ± 4.1	0.112
Chronic disease, *n*	30 (83.3)	15 (83.3)	15 (83.3)	> 0.999
Fall history, *n*	13 (36.1)	6 (33.3)	7 (38.9)	0.732
Frailty phenotype, *n*				0.573
Robust	17 (47.2)	10 (55.6)	7 (38.9)	
Pre-frail	16 (44.4)	7 (38.9)	9 (50.0)	
Frail	3 (8.3)	1 (5.6)	2 (11.1)	
MMSE, pt	28.2 ± 1.5	28.2 ± 1.7	28.3 ± 1.3	0.823
5-item GDS, pt	0 [0–1]	1 [0–1]	0.5 [0–1]	0.522
EQ-5D VAS, pt	72.7 ± 17.2	68.6 ± 18.7	76.8 ± 14.9	0.154
Short FES-I, pt	9.5 [8–11]	9.5 [8–11]	9.5 [8–11]	0.823
Treadmill experience, *n*	18 (50)	9 (50)	9 (50)	> 0.999
TUG, s	12.2 ± 4.8	12.2 ± 5.9	12.2 ± 3.6	0.968
SPPB, pt	9.9 ± 2.5	9.6 ± 2.8	10.3 ± 2.2	0.364
Gait speed, m/s^a^	0.84 ± 0.16	0.82 ± 0.20	0.86 ± 0.12	0.439
Daily step count	8435 [7742–8535]	8449 [7673–9168]	8406 [7962–8504]	0.752

Descriptive data given as mean ± standard deviation, median [interquartile range], or *n* (%). *P*-values calculated for* t*-tests for independent samples, Mann–Whitney *U* tests, and *χ*^*2*^-tests or Fisher’s exact tests

*6PBT* six-session perturbation-based balance training, *2PBT* two-session perturbation-based balance training and four-session conventional treadmill training, *BMI* body mass index, *MMSE* Mini-Mental State Examination, *GDS* Geriatric Depression Scale, *EQ-5D VAS* EuroQol-5 dimensions visual analogue scale, *Short FES-I* Short Falls Efficacy Scale-International, *TUG* Timed Up and Go, *SPPB* Short Physical Performance Battery

^a^based on the 4-m walk test of the SPPB

### Reactive balance

**Fig. 2:**
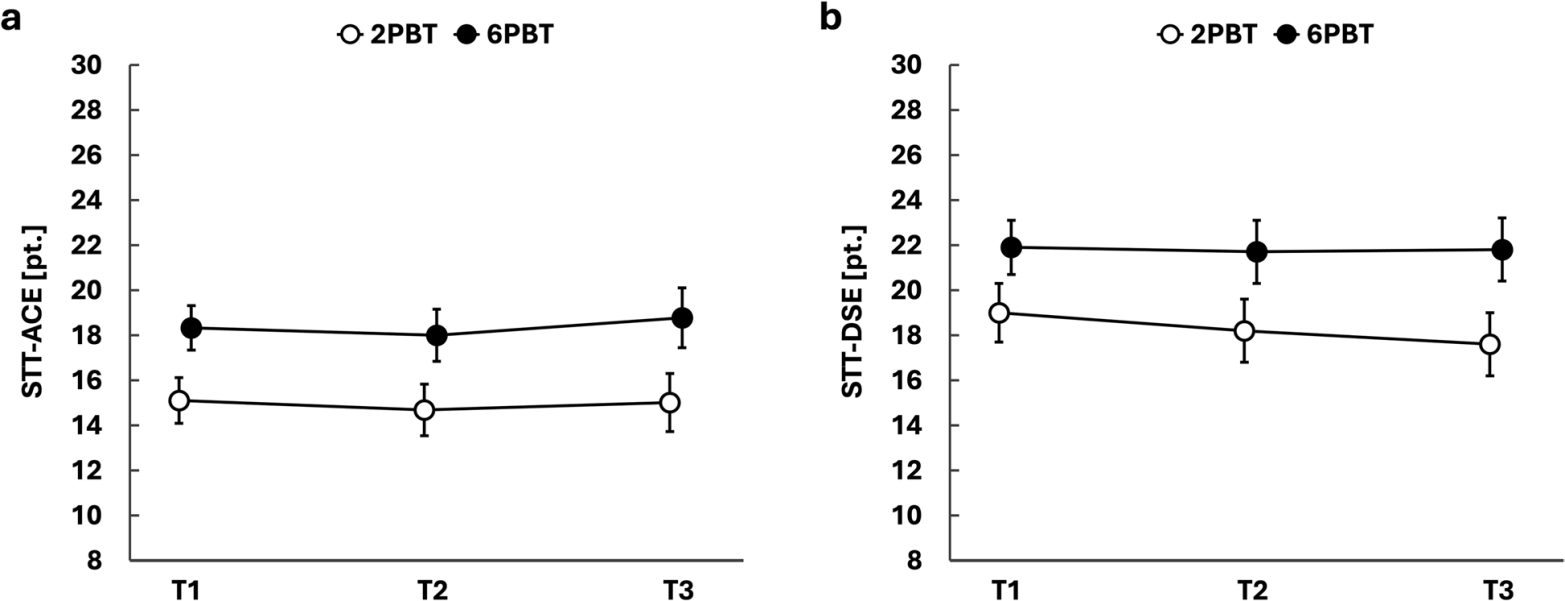
Between-group changes over time in static reactive balance. (a) STT-ACE = Stepping Threshold Test – all-step count evaluation, (b) STT-DSE = Stepping Threshold Test – direction-sensitive evaluation, 2PBT = two-session perturbation-based balance training and four-session conventional treadmill training, 6PBT = six-session perturbation-based balance training, T1 = baseline assessment, T2 = post-intervention assessment, T3 = follow-up assessment. Data given as estimated marginal means and standard errors

Repeated-measures ANOVA revealed no significant interaction between time and group for the STT-ACE (*p* = 0.779, *η*_*p*_^*2*^ = 0.008) and STT-DSE (*p* = 0.686, *η*_*p*_^*2*^ = 0.011), nor was there a significant main effect of time (STT-ACE: *p* = 0.562, *η*_*p*_^*2*^ = 0.018; STT-DSE: *p* = 0.566, *η*_*p*_^*2*^ = 0.018) (Table 2, Fig. 2).

**Table 2:** Effects of the two treadmill perturbation-based balance training protocols on reactive balance

Variable	T1	T2	T3	Time × Group		Time		Group	
				***p***	***η***_***p***_^**2**^	***p***	***η***_***p***_^**2**^	***p***	***η***_***p***_^**2**^
*Reactive balance*									
STT-ACE, pt									
6PBT	18.3 ± 1.0	18.0 ± 1.2	18.8 ± 1.3	0.779	0.008	0.562	0.018	0.011	0.172
2PBT	15.1 ± 1.0	14.7 ± 1.1	15.0 ± 1.3						
STT-DSE, pt									
6PBT	21.9 ± 1.2	21.7 ± 1.4	21.8 ± 1.4	0.686	0.011	0.566	0.018	0.022	0.149
2PBT	19.0 ± 1.3	18.2 ± 1.4	17.6 ± 1.4						
DSTT, pt									
6PBT	36.5 ± 6.8	64.8 ± 7.8^a^	63.8 ± 7.6^a,b^	0.027	0.101	0.008	0.132	0.068	0.097
2PBT	30.1 ± 6.8	46.5 ± 7.9^a^	37.3 ± 7.6^b^						

Descriptive data given as estimated marginal means ± standard errors, with *p*-values calculated for repeated-measures ANOVA (within-subject factor = time [T1, T2, T3], between-subject factor = group [6PBT, 2PBT]), adjusted for treadmill experience and gait speed to account for stratification variables

*T1* baseline assessment, *T2* post-intervention assessment, *T3* follow-up assessment, *STT-ACE* Stepping Threshold Test – all-step count evaluation, *STT-DSE* Stepping Threshold Test – direction-sensitive evaluation, *6PBT* six-session perturbation-based balance training, *2PBT* two-session perturbation-based balance training and four-session conventional treadmill training. Significant differences compared to ^a^T1 or ^b^the other PBT group in Bonferroni-corrected post-hoc tests

**Fig. 3:**
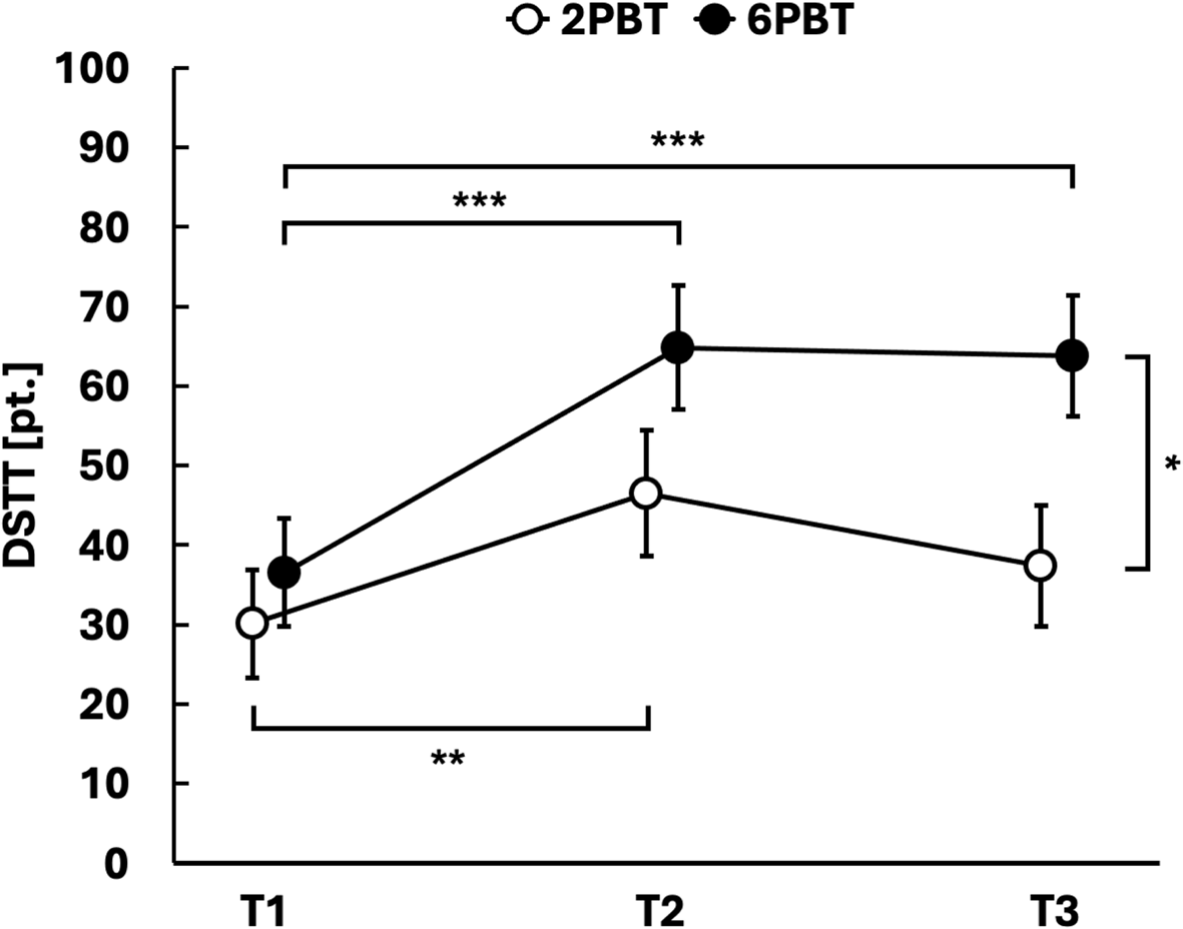
Between-group changes over time in dynamic reactive balance. DSTT = Dynamic Stepping Threshold Test, 2PBT = two-session perturbation-based balance training and four-session conventional treadmill training, 6PBT = six-session perturbation-based balance training, T1 = baseline assessment, T2 = post-intervention assessment, T3 = follow-up assessment. Data given as estimated marginal means and standard errors; *p < 0.05, **p < 0.01, ***p < 0.001 for Bonferroni-corrected post-hoc comparisons

A significant moderate interaction effect (*p* = 0.027, *η*_*p*_^*2*^ = 0.101) and a significant moderate time effect (*p* = 0.008, *η*_*p*_^*2*^ = 0.132) were observed for the DSTT. Post-hoc tests for multiple comparisons showed that both the 6PBT group (*p*_*Bonf*_ < 0.001) and the 2PBT group (*p*_*Bonf*_ = 0.005) significantly increased their DSTT score from T1 to T2, with no significant difference between groups at T2 (*p*_*Bonf*_ = 0.107). At T3, the 6PBT group maintained a significantly higher DSTT score compared to T1 (*p*_*Bonf*_ < 0.001), whereas the 2PBT group did not (*p*_*Bonf*_ = 0.211) resulting in a significantly higher DSTT score of 6PBT compared to the 2PBT group at T3 (*p*_*Bonf*_ = 0.015) (Table 2, Fig. 3).

Results of complete-case analysis were consistent with those of the primary multiple imputation analysis (Table S1 in Additional file 1). Full model outputs, including covariate main effects and within-subjects covariate interactions, are provided in Table S2 in Additional File 1.

### Secondary outcomes

No significant interaction effects were observed for secondary outcome measures on global and dynamic balance, gait capacity, functional mobility, PA, concerns about falling, and executive functioning (*p* = 0.066–0.723; *η*_*p*_^*2*^ = 0.010–0.074; Table 3). Significant moderate to large time effects were found for FSST, cadence, double support, TUG, mean daily step count, maximum step count per walking bout, Short FES-I, and TMT-A (*p* = 0.002–0.033; *η*_*p*_^*2*^ = 0.092–0.166). Post-hoc tests for multiple comparisons revealed significant improvements in the FSST, TUG, and maximum step count per walking bout from T1 to T2 (*p*_*Bonf*_ = 0.003–0.038), which were sustained at T3 compared to T1 (*p*_*Bonf*_ = 0.010–0.016). Cadence (*p*_*Bonf*_ = 0.004) and TMT-A (*p*_*Bonf*_ = 0.005) also significantly improved from T1 to T2, but these improvements were not sustained at T3 (cadence: *p*_*Bonf*_ = 0.063, TMT-A: *p*_*Bonf*_ = 0.098). Mean daily step count significantly decreased from T1 to T2 (*p*_Bonf_ = 0.007), but did not show significant differences between T1 and T3 (*p*_*Bonf*_ = 0.096). Double support (*p*_*Bonf*_ = 0.018) and Short FES-I (*p*_*Bonf*_ = 0.032) improved significantly from T1 to T3, but changes from T1 to T2 missed the level of significance (double support: *p*_*Bonf*_ = 0.092, Short FES-I: *p*_*Bonf*_ = 0.059).

**Table 3:** Effects of the two treadmill perturbation-based balance training protocols on secondary outcomes

Variable	T1	T2	T3	Time × Group		Time		Group	
				***p***	***η***_***p***_^**2**^	***p***	***η***_***p***_^**2**^	***p***	***η***_***p***_^**2**^
*Global balance*									
Brief-BESTest, pt									
6PBT	13.6 ± 1.2	12.7 ± 1.2	11.7 ± 1.2	0.066	0.074	0.576	0.016	0.297	0.031
2PBT	10.6 ± 1.2	11.3 ± 1.2	11.4 ± 1.2						
*Dynamic balance*									
FSST, s									
6PBT	13.0 ± 1.3	10.5 ± 0.8	11.0 ± 0.9	0.586	0.016	0.005	0.138	0.903	< 0.001
2PBT	12.4 ± 1.3	11.1 ± 0.8	10.9 ± 0.9						
*Gait capacity*									
Gait speed, m/s									
6PBT	0.99 ± 0.05	1.09 ± 0.08	1.07 ± 0.07	0.482	0.023	0.079	0.081	0.648	0.006
2PBT	1.03 ± 0.05	1.10 ± 0.09	1.07 ± 0.07						
Cadence, steps/min									
6PBT	110.7 ± 1.9	117.7 ± 2.5	115.7 ± 2.3	0.102	0.063	0.002	0.155	0.790	0.002
2PBT	114.7 ± 2.0	117.3 ± 2.6	114.2 ± 2.2						
Step time, s									
6PBT	0.55 ± 0.01	0.52 ± 0.02	0.53 ± 0.02	0.236	0.044	0.117	0.060	0.429	0.017
2PBT	0.53 ± 0.01	0.52 ± 0.03	0.53 ± 0.02						
Stride length, m									
6PBT	1.08 ± 0.05	1.12 ± 0.07	1.10 ± 0.07	0.447	0.024	0.211	0.047	0.696	0.005
2PBT	1.08 ± 0.05	1.14 ± 0.08	1.10 ± 0.07						
Double support, %									
6PBT	34.6 ± 1.6	32.4 ± 1.9	34.6 ± 1.4	0.504	0.020	0.033	0.092	0.309	0.030
2PBT	36.4 ± 1.5	35.5 ± 1.9	36.4 ± 1.4						
Walk ratio, cm/steps/min									
6PBT	0.49 ± 0.03	0.48 ± 0.04	0.48 ± 0.04	0.508	0.020	0.448	0.022	0.594	0.008
2PBT	0.47 ± 0.02	0.47 ± 0.04	0.49 ± 0.04						
2MWT, m									
6PBT	117.6 ± 7.8	115.1 ± 8.1	117.9 ± 9.1	0.246	0.040	0.674	0.011	0.423	0.019
2PBT	122.4 ± 7.8	127.7 ± 8.1	127.0 ± 9.1						
*Functional mobility*									
TUG, s									
6PBT	12.2 ± 1.2	11.7 ± 1.1	11.6 ± 1.0	0.151	0.053	0.002	0.166	0.520	0.012
2PBT	12.2 ± 1.2	10.2 ± 1.2	10.4 ± 1.0						
SPPB, pt									
6PBT	9.6 ± 0.6	10.0 ± 0.6	9.6 ± 0.6	0.327	0.032	0.421	0.025	0.425	0.018
2PBT	10.3 ± 0.6	10.3 ± 0.6	10.4 ± 0.6						
*Physical activity*									
Mean daily energy expenditure, METs									
6PBT	1.33 ± 0.07	1.43 ± 0.08	1.37 ± 0.08	0.340	0.031	0.351	0.031	0.486	0.014
2PBT	1.36 ± 0.06	1.37 ± 0.10	1.40 ± 0.09						
Mean daily duration in MVPA, min^a^									
6PBT	197.6 ± 19.6	200.1 ± 12.1	198.2 ± 16.6	0.332	0.032	0.762	0.008	0.397	0.021
2PBT	204.6 ± 16.6	183.6 ± 12.2	196.8 ± 16.8						
Mean daily step count^a^									
6PBT	8615 ± 458	7978 ± 353	8097 ± 467	0.684	0.011	0.033	0.096	0.967	< 0.001
2PBT	8323 ± 458	7534 ± 353	7976 ± 467						
Maximum step count per walking bout									
6PBT	83 ± 5	94 ± 5	91 ± 6	0.328	0.032	0.016	0.109	0.477	0.015
2PBT	80 ± 5	84 ± 5	90 ± 6						
*Concerns about falling*									
Short FES-I, pt									
6PBT	9.9 ± 0.6	9.5 ± 0.5	9.3 ± 0.5	0.723	0.010	0.026	0.097	0.355	0.025
2PBT	9.6 ± 0.6	8.8 ± 0.5	8.8 ± 0.5						
*Executive functioning*									
TMT-A, s									
6PBT	71.9 ± 8.3	61.2 ± 6.0	62.1 ± 7.3	0.650	0.013	0.019	0.105	0.535	0.011
2PBT	63.9 ± 8.3	54.0 ± 6.0	60.2 ± 7.3						
TMT-B, s									
6PBT	155.2 ± 17.4	155.5 ± 16.9	133.6 ± 13.2	0.674	0.011	0.106	0.066	0.733	0.003
2PBT	148.2 ± 17.4	149.2 ± 16.9	126.8 ± 13.2						

Descriptive data given as estimated marginal means ± standard errors, with *p*-values calculated for repeated-measures ANOVA (within-subject factor = time [T1, T2, T3], between-subject factor = group [6PBT, 2PBT])

*T1* baseline assessment, *T2* post-intervention assessment, *T3* follow-up assessment, *Brief-BESTest* Brief Balance Evaluation Systems Test, *6PBT* six-session perturbation-based balance training, *2PBT* two-session perturbation-based balance training and four-session conventional treadmill training, *FSST* Four-Square Step Test, *2MWT* 2-min walk test, *TUG* Timed Up and Go, *SPPB* Short Physical Performance Battery, *Short FES-I* Short Falls Efficacy Scale-International, *TMT* Trail Making Test

^a^Mean daily step count, mean daily MVPA duration, and TMT-B were analyzed after natural log-transformation due to non-normally distributed residuals

Significant moderate to large interaction and time effects were observed for the maximum perturbation magnitudes completed during the PBT sessions in the AP and ML directions (*p* < 0.001–0.034, *η*_*p*_^*2*^ = 0.118–0.733). Post-hoc tests for multiple comparisons revealed that both groups showed significant increases in AP and ML perturbation magnitudes from the first to the last PBT session (*p*_*Bonf*_ < 0.001), with the 6PBT group demonstrating significantly higher magnitudes than the 2PBT group at the last session (AP: *p*_*Bonf*_ = 0.007; ML: *p*_*Bonf*_ = 0.043) (Table S3 in Additional file 1).

The complete-case analysis showed similar results for maximum perturbation magnitudes (Table S3 in Additional file 1), double support and TUG, but missed significance for the time effect on PA outcomes, Short FES-I, and TMT-A, or for the post-hoc tests for the time effect on FSST and cadence (Table S4 in Additional File 1).

### Feasibility and acceptability

Nine participants (6PBT: *n* = 4, 22.2%; 2PBT: *n* = 5, 27.8%; *p* > 0.999) dropped out over the study period (Table 4, Fig. 1). Four (11.1%) withdrew after baseline assessment and before starting the intervention due to anxiety about receiving further perturbations, three (8.3%) dropped out during the intervention period due to medical reasons not directly related to the intervention. Two (5.5%) participants in the 6PBT group discontinued the intervention after 3 and 4 training sessions due to hip pain or anxiety about receiving further perturbations. However, they completed the post-intervention assessment but declined to participate in the follow-up assessment. No intervention-related serious adverse events were reported. Dropouts showed significantly fewer years of education (10.8 ± 3.8 vs. 13.9 ± 2.9 years, *p* = 0.016), a higher daily step count (10,393 [IQR 8453–10967] vs. 8310 [IQR 7708–8466] steps, *p* = 0.014), and more MVPA (305.3 [IQR 233.0–345.6] vs. 196.4 [IQR 157.6–216.8] min, *p* = 0.036) compared to study completers at T1, but there were no significant differences in other participant characteristics and outcomes (*p* = 0.265–0.918).

**Table 4:** Feasibility and acceptability of the perturbation-based treadmill training

Variable	6PBT (*n* = 18)	2PBT (*n* = 18)	*p*
Dropouts (total study period), *n*	4 (22.2)	5 (27.8)	> 0.999
Dropouts (intervention period), *n*	2 (11.1)	1 (5.6)	> 0.999
Adherence to PBT sessions, %^a^	100 [96.5–100.0]	100 [64.3–100.0]	0.505
Perturbations received, *n*^a^	221 [157.0–240.0]	78 [34.5–80.0]	< 0.001
Proportion of planned perturbations, %^a^	91.9 [67.5–100.0]	90.0 [43.1–100.0]	0.794
Acceptability, pt	26.8 ± 4.2	28.0 ± 5.7	0.474

Descriptive data given as *n* (%), median [interquartile range], and mean ± standard deviation

*P*-values calculated for Fisher’s exact tests (dropouts), Mann–Whitney *U* tests (adherence, perturbations received, proportion of planned perturbations), or *t*-tests for independent samples (acceptability)

*6PBT* six-session perturbation-based balance training, *2PBT* two-session perturbation-based balance training and four-session conventional treadmill training

^a^Participants who dropped out after the baseline assessment and before starting the intervention (*n* = 4) were recorded as having 0% adherence to PBT sessions and receiving 0 perturbations

The median PBT adherence rates were 100% [IQR 96.5–100.0] in the 6PBT group and 100% [IQR 64.3–100.0] in the 2PBT group, with no significant difference between groups (*p* = 0.505). The 6PBT group received on median 221 [IQR 157.0–240.0] perturbations during the intervention, while the 2PBT group received 78 [IQR 34.5–80.0] perturbations (*p* < 0.001). A high proportion of planned perturbations (median ≥ 90%) was observed in both groups, with no significant between-group difference (*p* = 0.794).

The acceptability of the PBT was also similarly high in both groups, with mean TFA questionnaire scores in the upper quartiles (6PBT = 26.8 ± 4.2 pt., 2PBT = 28.0 ± 5.7 pt.) and no significant between-group difference (*p* = 0.474). Complete-case analysis revealed similar findings (Additional file 1).

## Discussion

The FEATURE study investigated the dose–response relationship of treadmill PBT with different session numbers (six vs. two) for improving reactive balance and evaluated the feasibility and acceptability of the PBT protocols in older adults at risk of falling. To our knowledge, this study was the first to compare two multidirectional treadmill PBT protocols (AP and ML perturbations) with different dosages in this population. Since the dose–response relationship of PBT, especially in more frail older adults at risk of falling, is not yet fully understood, we examined two PBT protocols to explore the impact of perturbation frequency and to compare continuous weekly training (6PBT) with a “booster” training approach (2PBT) on immediate post-intervention effects and 6-week follow-up retention on reactive balance. Our hypothesis that 6PBT yields significantly greater improvements in reactive balance compared to 2PBT was partly confirmed. While both protocols led to task-specific improvements in reactive balance (DSTT) from baseline to post-intervention with no difference between them, only the 6PBT protocol showed sustained improvements at follow-up. This suggests that a higher PBT dose (i.e., more frequent and regular PBT sessions) may be necessary for achieving long-term gains in reactive balance among older adults at risk of falling. Feasibility and acceptability did not differ between protocols; both showed high training adherence, were well-accepted, and had no serious intervention-related adverse events.

### Efficacy on primary and secondary outcomes

Receiving a median of 78 perturbations over 6 weeks resulted in similar immediate post-intervention gains in dynamic reactive balance as receiving a median of 221 perturbations. This supports previous findings suggesting a non-linear dose–response relationship of PBT on reactive balance [21, 22], with no additional benefits of higher practice dosages (24 vs. 40 perturbations) [17] in high-functioning older adults. It also reinforces the idea of a critical practice dose required to provoke immediate adaptions, beyond which additional stimuli may provide no further benefits [52]. Similarly, our findings also suggest that in more physically frail older adults at risk of falling, a higher PBT dose (80 vs. 240 perturbations) did not yield greater immediate improvements. Future research is needed to determine whether even lower practice doses are sufficient to elicit immediate adaptions in this population.

Retention of dynamic reactive balance improvements 6 weeks after PBT cessation was observed only for the 6PBT protocol. These findings align with previous studies reporting also improved retention of reactive balance with higher practice doses of treadmill [55] or overground PBT [56] in older adults. It has been postulated that overlearning from higher PBT doses may enhance long-term retention by strengthening motor memory consolidation and facilitating retrieval [55–57]. While brief exposure to PBT may be sufficient for immediate reactive balance improvements, a critical practice dose appears also necessary for long-term retention. The lack of sustainable improvements in the 2PBT group suggests that the lower PBT dose may not have been sufficient to elicit overlearning. In contrast, the 6PBT group’s retention of reactive balance improvements indicates that the greater number of exposures (two-thirds more) may have been enough to consolidate motor memory for later retrieval.

Beyond the number of PBT sessions or perturbations, training dose can also be adjusted by perturbation intensity [8], which may also be crucial for retaining acute adaptions of reactive balance. Short-term (a few weeks) and long-term retention (up to 12 months) of reactive balance after single PBT sessions in older adults has been primarily reported in overground perturbation studies, where consistently high-magnitude perturbations were applied [56, 58, 59]. Due to the participants’ limited physical capacity, we used a progressive increase in perturbation magnitude, beginning with lower-magnitude perturbations and progressing based on their self-perceived difficulty and anxiety levels to enhance tolerance, build confidence, and reduce dropout, as previously recommended for more frail older populations [8, 60]. This progressive approach may, however, require a greater total number of perturbations to reach the critical practice dose of high-magnitude perturbations needed for retention, described as a “rightward shift in the practice dose–response relationship for perturbation training” [23]. Indeed, the 2PBT group reached significantly lower maximum perturbation magnitudes than the 6PBT group in the last PBT session, while perturbation magnitudes in the first PBT session were similar. This suggests that the 2PBT protocol was not sufficient to reach the minimum perturbation practice dose required for long-term retention in terms of both the perturbation number and intensity.

The effects of PBT on reactive balance have been shown to wane over time [56]. Compared to a single PBT session, an ancillary booster session of PBT provided three months after an initial PBT session has been found to reduce decay and support retention of PBT-specific improvements in reactive balance for up to six months among high-functioning older adults [56]. Although our study did not allow for a direct comparison with a single PBT session, the booster session in the 2PBT group, administered five weeks after the initial PBT session, yielded post-intervention benefits on dynamic reactive balance similar to those observed for the 6PBT protocol. This suggest that the second PBT session may have contributed to maintaining PBT-specific effects over shorter periods to a level comparable to continuous PBT during the same period. However, reactive balance improvements in the 2PBT group were not sustained approximately three months after the initial PBT session. This indicates that shorter intervals between PBT sessions, potentially less than six weeks, may be necessary to sustain improvements when reactive balance is initially developed with fewer PBT sessions. Whether longer retention intervals can be achieved with a higher initial PBT dose remains an open question for future research.

Improvements observed in dynamic (DSTT) but not in static reactive balance (STT) suggest that PBT is highly task-specific, as participants were exposed to solely gait perturbations during the intervention period. Previous studies on the generalization of PBT in older adults without mobility limitations have reported mixed results [17, 37, 61–63]. Some showed positive transfer across different contexts of the same perturbation type, such as from treadmill-gait slips to overground-gait slips [17] or from overground gait-slips simulated with a moveable platform to gait-slips on an actual slippery surface [61]. In contrast, other studies could not document such transfer, whether between different contexts with the same perturbation type (from treadmill gait-trips to overground gait-trips) [37], between different perturbation types within the same context (from overground gait-slips to overground gait-trips) [62], or across different motor tasks involving the same perturbation type (from standing perturbations to gait perturbations) [63]. Our findings contribute to these PBT studies on non-generalizability by showing a lack of transfer of improved reactive balance skills not only from gait to standing perturbations but also among more frail older adults. This also supports the concept of high task-specificity in balance training, suggesting that the ability to maintain balance across diverse tasks relies more on the accumulation of specifically learned skills than on a general capacity that can be improved irrespective of the trained task [64].

Beneficial time effects were observed for dynamic balance, gait capacity, functional mobility, PA, concerns about falling, and executive functioning, with no between-group differences over time. These findings indicate that both intervention arms – regardless of the number of PBT sessions – may have been effective in improving these secondary outcomes. PBT did not appear to affect other non-reactive physical capacity measures, PA, or psychological and cognitive functioning in our study; rather the improvements in these outcomes may have been driven by treadmill walking itself. However, as both groups received treadmill walking with different PBT session numbers and there was not a further group performing only CTT, it is challenging to isolate the unique effects of PBT versus treadmill walking. For instance, previous studies have shown that treadmill training without surface perturbations can improve physical capacity [65–68], concerns about falling [69], and executive functioning [69] in older adults. In addition, other studies comparing treadmill PBT to CTT have reported mixed results. Some found superior effects of PBT on physical capacity [70] and concerns about falling [11], while others found no such effects on physical capacity [70–72], psychological [70–72], and cognitive functioning [71].

The absence of significant effects on the Brief-BESTest is consistent with previous studies reporting also no beneficial effects of treadmill PBT on global balance measures (Brief-BESTest [19], Mini-BESTest [73], Berg Balance Scale [28]). Given the task-specific nature of PBT, with limited generalization to non-perturbed and less dynamic or static balance tasks [8], and the fact that the Brief-BESTest does not specifically assess dynamic reactive balance [41], this result is not surprising.

The significant decrease in mean daily step count during the intervention period, with no between-group differences, may reflect activity compensation, as participants adjusted their non-structured daily activity to maintain overall usual PA and energy expenditure, consistent with the “activitystat hypothesis” [74]. The treadmill sessions, integrated into the once-weekly training at REGE e.V., were likely more intensive than the usual balance and strength exercises, potentially leading to reduced non-structured PA, which returned to levels similar to baseline after the intervention.

Notably, maximum step count per walking bout, a capacity-related PA outcome, showed a sustainable increase, with also no between-group differences. This improvement likely stemmed from training characteristics in both PBT and CTT, such as uninterrupted treadmill walking for 1.5 to 3.5 min, which may have enhanced the ability to sustain prolonged walking bouts in daily life. Improvements in rhythm-related (cadence, double support) but not pace-related gait parameters (2MWT, gait speed) may be attributed to the constant treadmill speed used throughout the intervention and the focus on progressively increasing perturbation magnitude to improve reactive balance. These specific training characteristics may have limited adaptations in pace-related parameters while fostering gains in rhythm-related parameters, which are more directly associated with balance control and have been identified as preferred compensatory strategies to enhance stability during challenging locomotor tasks in older adults [75].

### Feasibility and acceptability

In addition to exploring the dose–response relationship of PBT, there also remains a need for feasibility studies to identify facilitators and barriers to its implementation, as well as strategies to alleviate anxiety in participants undergoing PBT to ensure its practicality [8]. To address this research gap, this study also examined the feasibility and acceptability of PBT in older adults at risk of falling.

The dropout rate showed no between-group differences and was 25% overall, with less than 10% among participants who started the PBT. The total dropout rate was higher than expected (15%) [33]. This may be attributed to the more challenging nature of PBT and reactive balance assessments, compared to exercise interventions and physical capacity assessments conducted in the same setting (REGE e.V.) with a comparable study population [51, 52],

Indeed, four participants (11%) dropped out after baseline assessment, which included the STT and DSTT, due to anxiety about receiving further perturbations on the treadmill during the study period. Comparison of these dropouts with participants who started the PBT did not suggest any differences in sociodemographic, physical, psychological, cognitive or other (e.g., treadmill experience, fall history) baseline characteristics (data not shown). Anxiety-related dropouts have also been previously reported among high-functioning older adults receiving perturbations during treadmill [73] or overground walking [16]. Both the STT and DSTT aimed to specifically assess participants’ limits of reactive balance capacity. Such assessments at baseline may intimidate participants and heighten anxiety about subsequent PBT. To prevent dropouts before PBT participation, potential strategies could include submaximal perturbation-based reactive balance assessments or omitting these assessments at baseline in RCTs, assuming successful randomization with similar reactive balance capacity levels across study arms. Performing these assessments only during follow-ups in RCTs would also ensure that “first-trial” effects of PBT [59] are eliminated in the study arms not intended to receive perturbations. Additionally, other studies have reported that older adults who were initially anxious often found their anxiety diminished or resolved after experiencing progressive perturbations during initial PBT sessions and gaining confidence in their ability to recover from perturbations [30, 76]. Therefore, we recommend implementing such strategies to ensure that more anxious individuals engage in and can benefit from PBT interventions.

During the intervention period, only one participant (2.8%) interrupted the PBT due to anxiety about receiving further perturbations. This low number of anxiety-related dropouts in those participants starting the PBT is likely related to our approach of involving participants in determining training intensity and progression by considering and monitoring their self-perceived anxiety and difficulty levels during PBT. Such approach has been associated with none or very low (< 5%) anxiety-related dropouts during treadmill [14, 73] and overground PBT [16] in high-functioning older adults, and has also been recommended from therapists using PBT in daily practice to enhance overall PBT experience for participants [76].

Successive progression from low- to high-magnitude perturbations may have further contributed to preventing PBT-related dropouts and maintaining training adherence [8, 60]. However, this approach may have slightly compromised the efficacy of some perturbations, as they did not meet the strict definition of PBT.

Adherence rates to the PBT sessions were high and comparable to other treadmill PBT in higher-functioning older adults [14, 19, 73], or other fall prevention exercise programs for older adults [77].

The proportion of planned perturbations completed was also high. In those participants starting the interventions, 87% (4706 out of 5440) of planned perturbations could be applied. Two main reasons accounted for incomplete perturbations aside from dropouts. First, despite 50% having prior treadmill experience, walking on the perturbation treadmill without handrails was challenging for some participants, requiring trainers to provide grasp support during initial sessions, as also reported in another PBT study using the same perturbation treadmill among (pre-)frail geriatric patients [20]. This led to skipping or replacing perturbation blocks for familiarization with handrail-free treadmill walking. To address this, we recommend an ancillary familiarization session to accommodate participants to treadmill walking before starting treadmill PBT, as done in previous studies [19, 24]. Alternatively, pre-tests could be used to screen for sufficient treadmill walking ability, which has been shown to significantly reduce dropouts in (pre-)frail geriatric patients [20]. Second, fatigue from treadmill walking – rather than the perturbations themselves – caused some participants to prematurely terminate or skip training blocks. For example, in participants with the most impaired gait patterns, the perturbation treadmill required longer durations to detect the specific gait swing phase in which the perturbation was induced. This increased the time intervals between individual perturbations, contributing to overall fatigue. Adjustments such as shorter training blocks (e.g., a maximum of 2.5 min) with reduced wash-out times between perturbations (e.g., 7–15 s) could help deliver the same number of perturbations within a shorter training duration, minimizing fatigue and improving adherence.

Acceptability of PBT in older adults remains understudied, and comparisons of PBT protocols with different number of PBT sessions has not yet been conducted. Based on the TFA questionnaire, we found that the acceptability of treadmill PBT was not affected by the number of PBT sessions received and was generally favorable in our sample. This aligns with other quantitative [32] and qualitative studies on treadmill PBT [30, 31], which concluded that treadmill PBT is acceptable among older adults. Notably, our findings also highlight the acceptability of treadmill PBT in more physically frail older adults.

## Limitations

Some limitations of this study warrant consideration. First, due to its pilot nature, the sample size was small, limiting the generalization of the findings. It also precluded analyzing the effects of the PBT protocols on daily-life falls because of insufficient statistical power for this outcome. Future large-scale, definitive RCTs are needed to confirm our findings and to assess the dose–response relationship of PBT on fall incidences in daily life among older adults at risk of falling. Second, the participants were recruited from a senior fitness club, which likely limits the generalization of findings to older adults who do not regularly engage in structured physical exercise. Third, while the DSTT is a modified version of the validated STT, its psychometric properties are still unknown. Moderate to high correlations with other reactive, dynamic and global balance (STT, FSST, Brief-BESTest: *rho* = 0.56–0.76), gait capacity (gait speed: *rho* = 0.47; 2MWT: *rho* = 0.69), and functional mobility measures at baseline (TUG: *rho* = −0.62, SPPB: *rho* = 0.41) suggest some initial convergent validity. However, further studies are needed to establish the psychometric properties of the DSTT. Fourth, although the progressive increase in perturbation magnitude in the STT and DSTT to reach each participant’s limit of reactive balance control likely minimized a first-trial effect, its influence cannot be fully excluded. Fifth, acceptability was assessed using a self-designed, non-validated questionnaire based on the TFA, as the recently validated generic TFA questionnaire [78] was not yet published or available at the start of the study. Sixth, the complete-case analysis did not confirm all findings for some secondary outcomes observed in the primary multiple imputation analysis, which limits the robustness of these findings and underscores the need for cautious interpretation. Finally, the study design included only a 6-week follow-up, providing insights into the short-term sustainability but leaving long-term effects uncertain.

## Conclusions

Our findings support recent evidence that even a low number of treadmill PBT sessions can lead to task-specific improvements in reactive balance during walking among older adults at risk of falling, while a higher PBT practice dose may enhance the sustainability of such improvements. Treadmill PBT appears to be safe, feasible, and acceptable in this population, regardless of the number of sessions received, when PBT progression accounts for individuals’ subjective difficulty and anxiety levels. For implementation of treadmill PBT in more frail older adults at risk of falling, we recommend (1) pre-screening for treadmill walking ability and/or a sufficient ancillary familiarization phase to help participants to adapt to unperturbed treadmill walking without grasp support, (2) using short training blocks to prevent PBT session interruptions due to fatigue associated with treadmill walking, and (3) avoiding initial reactive balance assessments with high-magnitude perturbations on the same treadmill used for subsequent PBT to minimize anxiety about the treadmill and perturbations and prevent early dropouts.

## Supplementary Information

Supplementary Material 1.

## Data Availability

The datasets used and/or analyzed during the current study are available from the corresponding author on reasonable request.

## Abbreviations

2MWT: 2-min walk test
2PBT: Two-session perturbation-based balance training and four-session conventional treadmill training
6PBT: Six-session perturbation-based balance training
ACE: All-step count evaluation
ANOVA: Analysis of variance
AP: Anterior–posterior
BESTest: Balance Evaluation Systems Test
CTT: Conventional treadmill training
DSTT: Dynamic Stepping Threshold Test
DSE: Dynamic-sensitive evaluation
FES-I: Falls Efficacy Scale-International
FSST: Four-Square Step Test
MET: Metabolic equivalent of tasks
ML: Medio-lateral
MMSE: Mini-Mental State Examination
MVPA: Moderate-to-vigorous physical activity
PA: Physical Activity
PBT: Perturbation-based balance training
REGE: Rehabilitation Sports in Geriatrics
RCT: Randomized controlled trial
SPPB: Short Physical Performance Battery
STT: Stepping Threshold Test
T1: Baseline assessment
T2: Post-intervention assessment
T3: Follow-up assessment
TFA: Theoretical Framework of Acceptability
TUG: Timed Up and Go Test
