# Supplementary material for: Dose–response relationship of treadmill perturbation-based balance training for improving reactive balance in older adults at risk of falling: results of the FEATURE randomized controlled pilot trial
Source: Eur Rev Aging Phys Act. 2025 May 16;22:8. doi: 10.1186/s11556-025-00375-w (PMC12082977; doi:10.1186/s11556-025-00375-w)
Supplement: Supplementary file 1 — Supplementary Material 1. [file 11556_2025_375_MOESM1_ESM.docx]

**Additional file 1**

**Table S1** Effects of the two treadmill perturbation-based balance training protocols on reactive balance (complete-case analysis)

| **Variable** | **T1** | **T2** | **T3** | **Time × Group** | |  | **Time** | |  | **Group** | |
| --- | --- | --- | --- | --- | --- | --- | --- | --- | --- | --- | --- |
|  |  |  |  | ***p*** | ***η_p_*^2^** |  | ***p*** | ***η_p_*^2^** |  | ***p*** | ***η_p_*^2^** |
| *Reactive balance* | | | | | | | | | | | |
| STT-ACE, pt. | | | | | | | | | | | |
| 6PBT (*n* = 12)^†^ | 18.5 ± 1.2 | 18.9 ± 1.4 | 19.8 ± 1.6 | 0.783 | 0.014 |  | 0.502 | 0.038 |  | 0.059 | 0.184 |
| 2PBT (*n* = 10)^#^ | 15.0 ± 1.3 | 15.2 ± 1.5 | 15.3 ± 1.8 |  |  |  |  |  |  |  |  |
| STT-DSE, pt. | | | | | | | | | | | |
| 6PBT (*n* = 12)^†^ | 21.9 ± 1.5 | 21.7 ± 1.6 | 21.8 ± 1.5 | 0.821 | 0.011 |  | 0.620 | 0.026 |  | 0.072 | 0.168 |
| 2PBT (*n* = 10)^#^ | 19.2 ± 1.6 | 18.9 ± 1.8 | 18.0 ± 1.7 |  |  |  |  |  |  |  |  |
| DSTT, pt. | | | | | | | | | | | |
| 6PBT (*n* = 12)^†^ | 43.3 ± 9.3 | 73.8 ± 9.9^a^ | 71.5 ± 10.6^a,b^ | 0.016 | 0.215 |  | 0.104 | 0.125 |  | 0.122 | 0.135 |
| 2PBT (*n* = 10)^#^ | 33.4 ± 9.8 | 50.5 ± 10.4^a^ | 37.4 ± 11.1^b^ |  |  |  |  |  |  |  |  |
| Descriptive data given as estimated marginal means ± standard errors, with *p*-values calculated for repeated-measures ANOVA (within-subject factor = time [T1, T2, T3], between-subject factor = group [6PBT, 2PBT]), adjusted for treadmill experience and gait speed to account for stratification variables.  ^†^Six participants not included in the complete case analysis due to dropout during the intervention period caused by unplanned eye surgery (*n* = 1) or acute health deterioration (*n* = 1), and unwillingness to complete the follow-up assessment (*n* = 2) or reactive balance assessment at T3 (*n* = 2).  ^#^Eight participants not included in the complete-case analysis due to dropout after the baseline assessment caused by anxiety about receiving further perturbations during the study period (*n* = 4), dropout during the intervention period caused by an intervention-unrelated injurious fall (*n* = 1), anxiety-related refusal to complete the reactive balance assessment at T1 (*n* = 1), or unwillingness to perform the reactive balance assessment at T3 (*n* = 2).  T1 = baseline assessment, T2 = post-intervention assessment, T3 = follow-up assessment, STT-ACE = Stepping Threshold Test – all-step count evaluation, STT-DSE = Stepping Threshold Test – direction-sensitive evaluation, DSTT = Dynamic Stepping Threshold Test, 6PBT = six-session perturbation-based balance training, 2PBT = two-session perturbation-based balance training and four-session conventional treadmill training. Significant differences compared to ^a^T1 or ^b^the other PBT group in Bonferroni-corrected post-hoc tests. | | | | | | | | | | | |

| a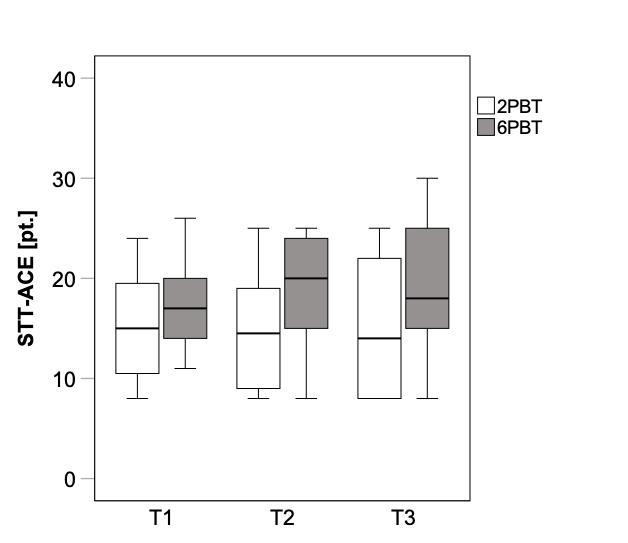 | b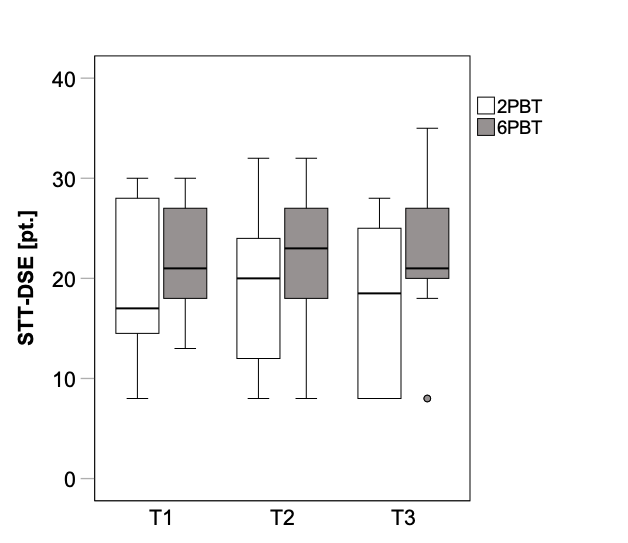 | c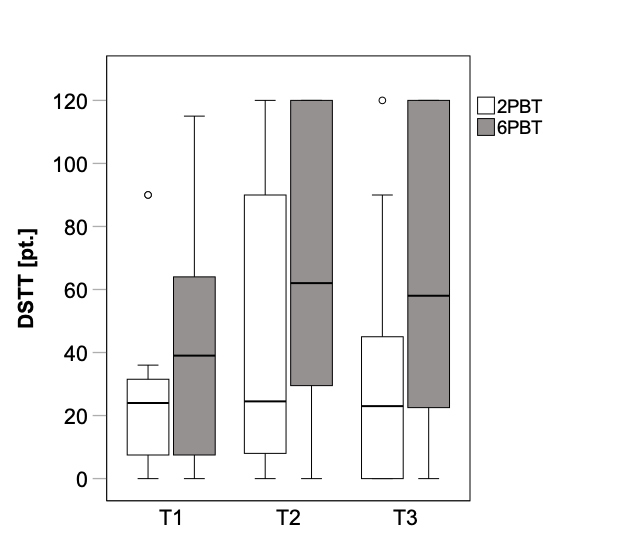 |
| --- | --- | --- |

**Fig. S1.** Boxplots for the primary outcomes of the 6PBT group (*n*=12; grey) and 2PBT group (*n*=10; white) at the baseline (T1), post-intervention (T2), and follow-up (T3) assessments. (a) Stepping Threshold Test – all-step count evaluation (STT-ACE), (b) Stepping Threshold Test – direction-sensitive evaluation (STT-DSE), and (c) Dynamic Stepping Threshold Test (DSTT). Data given for the complete-case analysis.

**Reactive balance**

The complete-case analysis revealed no significant interaction or time effects for the STT-ACE and STT-DSE (*p*=0.502-0.821; Table S1). However, a significant large interaction effect was observed for the DSTT (*p*=0.016, *η_p_^2^*=0.215). Post-hoc tests indicated that both the 6PBT group (*p_Bonf_*<0.001) and the 2PBT group (*p_Bonf_*=0.046) significantly improved their DSTT score from T1 to T2, with no significant difference between groups at T2 (*p_Bonf_*=0.125). At T3, the 6PBT group maintained a significantly higher DSTT score compared to T1 (*p_Bonf_*<0.001), whereas the 2PBT group did not (*p_Bonf_*=0.095). In addition, the 6PBT group showed a significantly higher DSTT score compared to the 2PBT group at T3 (*p_Bonf_*=0.041).

**Table S2** Full outputs from repeated-measures analyses of variance with main effects, covariate effects, and interactions for the multiple imputation analysis and complete-case analysis

| **Variable** | **Effect** | **Multiple imputation analysis** | |  | **Complete-case analysis** | |
| --- | --- | --- | --- | --- | --- | --- |
|  |  | ***p*** | ***η_p_*^2^** |  | ***p*** | ***η_p_*^2^** |
| STT-ACE | *Primary interaction* |  |  |  |  |  |
|  | Time × Group | 0.779 | 0.008 |  | 0.783 | 0.014 |
|  | *Main effects* |  |  |  |  |  |
|  | Time | 0.562 | 0.018 |  | 0.502 | 0.038 |
|  | Group | 0.011 | 0.172 |  | 0.059 | 0.184 |
|  | *Covariate effects* |  |  |  |  |  |
|  | Treadmill experience | 0.400 | 0.021 |  | 0.631 | 0.013 |
|  | Gait speed | <0.001 | 0.310 |  | <0.001 | 0.527 |
|  | *Within-subjects* *covariate interactions* |  |  |  |  |  |
|  | Time × treadmill experience | 0.078 | 0.074 |  | 0.113 | 0.114 |
|  | Time × gait speed | 0.715 | 0.010 |  | 0.978 | 0.001 |
| STT-DSE | *Primary interaction* |  |  |  |  |  |
|  | Time × Group | 0.686 | 0.011 |  | 0.821 | 0.011 |
|  | *Main effects* |  |  |  |  |  |
|  | Time | 0.566 | 0.018 |  | 0.620 | 0.026 |
|  | Group | 0.022 | 0.149 |  | 0.072 | 0.168 |
|  | *Covariate effects* |  |  |  |  |  |
|  | Treadmill experience | 0.464 | 0.014 |  | 0.631 | 0.013 |
|  | Gait speed | <0.001 | 0.392 |  | <0.001 | 0.527 |
|  | *Within-subjects* *covariate interactions* |  |  |  |  |  |
|  | Time × treadmill experience | 0.233 | 0.044 |  | 0.113 | 0.114 |
|  | Time × gait speed | 0.624 | 0.015 |  | 0.978 | 0.001 |
| DSTT | *Primary interaction* |  |  |  |  |  |
|  | Time × Group | 0.027 | 0.101 |  | 0.016 | 0.215 |
|  | *Main effects* |  |  |  |  |  |
|  | Time | 0.008 | 0.132 |  | 0.104 | 0.125 |
|  | Group | 0.068 | 0.097 |  | 0.122 | 0.135 |
|  | *Covariate effects* |  |  |  |  |  |
|  | Treadmill experience | 0.157 | 0.057 |  | 0.143 | 0.122 |
|  | Gait speed | <0.001 | 0.292 |  | 0.016 | 0.294 |
|  | *Within-subjects* *covariate interactions* |  |  |  |  |  |
|  | Time × treadmill experience | 0.593 | 0.016 |  | 0.664 | 0.024 |
|  | Time × gait speed | <0.001 | 0.219 |  | 0.002 | 0.307 |
| STT-ACE = Stepping Threshold Test – all-step count evaluation, STT-DSE = Stepping Threshold Test – direction-sensitive evaluation, DSTT = Dynamic Stepping Threshold Test. | | | | | | |

**Table S3** Maximum perturbation magnitude completed at the first and last treadmill perturbation-based balance training session

| **Variable** | **First PBT**  **session** | **Last PBT**  **session** | **Time × Group** | |  | **Time** | |  | **Group** | |
| --- | --- | --- | --- | --- | --- | --- | --- | --- | --- | --- |
|  |  |  | ***p*** | ***η_p_*^2^** |  | ***p*** | ***η_p_*^2^** |  | ***p*** | ***η_p_*^2^** |
| Multiple imputation analysis | | | | | | | | | | |
| *AP perturbation magnitude* | | | | | | | | | | |
| 6PBT (*n* = 18) | 12.0 ± 1.3 | 22.3 ± 1.6^a,b^ | 0.003 | 0.218 |  | <0.001 | 0.733 |  | 0.047 | 0.104 |
| 2PBT (*n* = 18) | 10.8 ± 1.3 | 16.1 ± 1.6^a,b^ |  |  |  |  |  |  |  |  |
| *ML perturbation magnitude* | | | | | | | | | | |
| 6PBT (*n* = 18) | 11.3 ± 1.2 | 20.6 ± 1.8^a,b^ | 0.034 | 0.118 |  | <0.001 | 0.614 |  | 0.090 | 0.078 |
| 2PBT (*n* = 18) | 10.3 ± 1.2 | 15.4 ± 1.8^a,b^ |  |  |  |  |  |  |  |  |
| Complete-case analysis | | | | | | | | | | |
| *AP perturbation magnitude* | | | | | | | | | | |
| 6PBT (*n* = 14)^†^ | 12.7 ± 1.5 | 23.2 ± 2.0^a,b^ | <0.001 | 0.395 |  | <0.001 | 0.740 |  | 0.045 | 0.151 |
| 2PBT (*n* = 13)^#^ | 11.1 ± 1.6 | 14.8 ± 2.0^a,b^ |  |  |  |  |  |  |  |  |
| *ML perturbation magnitude* | | | | | | | | | | |
| 6PBT (*n* = 14)^†^ | 11.9 ± 1.4 | 21.3 ± 2.2^a,b^ | 0.020 | 0.197 |  | <0.001 | 0.572 |  | 0.101 | 0.104 |
| 2PBT (*n* = 13)^#^ | 10.6 ± 1.5 | 14.4 ± 2.3^a,b^ |  |  |  |  |  |  |  |  |
| Descriptive data given as means ± standard errors, with *p*-values calculated for repeated-measures ANOVA (within-subject factor = time [first PBT session, last PBT session], between-subject factor = group [6PBT, 2PBT]). AP = anterior-posterior, ML = mediolateral; 6PBT = six-session perturbation-based balance training, 2PBT = two-session perturbation-based balance training and four-session conventional treadmill training;  ^†^Four participants not included in the complete-case analysis as they did not complete the sixth perturbation-based balance training session due to dropout during the intervention period caused by unplanned eye surgery (*n* = 1) or acute health deterioration (*n* = 1), or unwillingness (*n* = 2).  ^#^Five participants not included in the complete-case analysis due to dropout after the baseline assessment caused by anxiety about receiving further perturbations during the study period (*n* = 4) or dropout during the intervention period caused by an intervention-unrelated injurious fall (*n* = 1). Significant differences compared to ^a^T1 or ^b^the other PBT group in Bonferroni-corrected post-hoc tests. | | | | | | | | | | |

**Maximum perturbation magnitudes in PBT sessions**

The complete case analysis revealed significant large interaction and time effects for the maximum perturbation magnitudes applied during the PBT sessions (*p*<0.001-0.020, *η_p_²*=0.197-0.740). Post-hoc tests revealed that both groups showed significant increases in perturbation magnitudes from the first to the last PBT session in AP (6PBT:*p_Bonf_*<0.001, 2PBT: *p_Bonf_*=0.005) and ML (6PBT: *p_Bonf_*<0.001, 2PBT: *p_Bonf_*=0.031) directions, with the 6PBT group demonstrating significantly higher magnitudes than the 2PBT group at the last PBT session (AP: *p_Bonf_*=0.006; ML: *p_Bonf_*=0.037).

**Table S4** Effects of the two treadmill perturbation-based balance training protocols on secondary outcomes (complete case analysis)

| **Variable** | **T1** | **T2** | **T3** | **Time × Group** | |  | **Time** | |  | **Group** | | |
| --- | --- | --- | --- | --- | --- | --- | --- | --- | --- | --- | --- | --- |
|  |  |  |  | ***p*** | ***η_p_*^2^** |  | ***p*** | ***η_p_*^2^** |  | ***p*** | ***η_p_*^2^** | |
| *Global Balance* | | | | | | | | | | | |  |
| Brief-BESTest, pt. | | | | | | | | | | | |  |
| 6PBT (*n* = 13)^‡^ | 12.8 ± 1.5 | 13.0 ± 1.5 | 12.3 ± 1.6 | 0.510 | 0.029 |  | 0.930 | 0.003 |  | 0.432 | 0.027 | |
| 2PBT (*n* = 12)^#^ | 10.8 ± 1.4 | 11.0 ± 1.4 | 11.4 ± 1.5 |  |  |  |  |  |  |  |  | |
| *Dynamic balance* | | | | | | | | | | | |  |
| FSST, s | FSST, s |  |  |  |  |  |  |  |  |  |  | |
| 6PBT (*n* = 12)^§^ | 11.8 ± 1.4 | 10.8 ± 1.1 | 10.0 ± 1.0 | 0.778 | 0.007 |  | 0.030 | 0.165 |  | 0.732 | 0.005 | |
| 2PBT (*n* = 13)^¶^ | 12.1 ± 1.3 | 11.3 ± 1.1 | 10.9 ± 1.0 |  |  |  |  |  |  |  |  | |
| *Gait capacity* | | | | | | | | | | | |  |
| Gait speed, m/s | | | | | | | | | | | |  |
| 6PBT (*n* = 13)^$^ | 0.97 ± 0.06 | 1.10 ± 0.08 | 1.07 ± 0.06 | 0.553 | 0.021 |  | 0.007 | 0.217 |  | 0.788 | 0.003 | |
| 2PBT (*n* = 13)^¶^ | 1.03 ± 0.06 | 1.12 ± 0.08 | 1.06 ± 0.06 |  |  |  |  |  |  |  |  | |
| Cadence, steps/min | | | | | | | | | | | |  |
| 6PBT (*n* = 13)^$^ | 110.6 ± 2.4 | 117.4 ± 3.5 | 116.0 ± 3.1 | 0.293 | 0.050 |  | 0.029 | 0.138 |  | 0.890 | 0.001 | |
| 2PBT (*n* = 13)^¶^ | 114.2 ± 2.4 | 117.2 ± 3.5 | 114.1 ± 3.1 |  |  |  |  |  |  |  |  | |
| Step time, s | | | | | | | | | | | |  |
| 6PBT (*n* = 13)^$^ | 0.55 ± 0.01 | 0.52 ± 0.02 | 0.52 ± 0.02 | 0.228 | 0.060 |  | 0.034 | 0.131 |  | 0.800 | 0.003 | |
| 2PBT (*n* = 13)^¶^ | 0.53 ± 0.01 | 0.52 ± 0.02 | 0.53 ± 0.02 |  |  |  |  |  |  |  |  | |
| Stride length, m | | | | | | | | | | | |  |
| 6PBT (*n* = 13)^$^ | 1.06 ± 0.06 | 1.12 ± 0.07 | 1.11 ± 0.06 | 0.796 | 0.006 |  | 0.004 | 0.236 |  | 0.848 | 0.002 | |
| 2PBT (*n* = 13)^¶^ | 1.08 ± 0.06 | 1.15 ± 0.07 | 1.12 ± 0.06 |  |  |  |  |  |  |  |  | |
| Double support, % | | | | | | | | | | | |  |
| 6PBT (*n* = 13)^$^ | 35.2 ± 2.0 | 32.2 ± 2.4 | 34.4 ± 1.9 | 0.451 | 0.033 |  | 0.016 | 0.157 |  | 0.371 | 0.034 | |
| 2PBT (*n* = 13)^¶^ | 36.9 ± 2.0 | 36.1 ± 2.4 | 34.5 ± 1.9 |  |  |  |  |  |  |  |  | |
| Walk ratio, cm/steps/min | | | | | | | | | | | |  |
| 6PBT (*n* = 13)^$^ | 0.48 ± 0.03 | 0.48 ± 0.03 | 0.49 ± 0.03 | 0.234 | 0.059 |  | 0.172 | 0.071 |  | 0.960 | <0.001 | |
| 2PBT (*n* = 13)^¶^ | 0.47 ± 0.03 | 0.49 ± 0.03 | 0.50 ± 0.03 |  |  |  |  |  |  |  |  | |
| 2MWT, m | | | | | | | | | | | |  |
| 6PBT (*n* = 14)^^^ | 114.0 ± 9.5 | 111.4 ± 10.5 | 115.3 ± 11.9 | 0.497 | 0.028 |  | 0.816 | 0.008 |  | 0.324 | 0.039 | |
| 2PBT (*n* = 13)^¶^ | 127.5 ± 9.9 | 130.0 ± 10.9 | 128.9 ± 12.3 |  |  |  |  |  |  |  |  | |
| *Functional mobility* | | | | | | | | | | | |  |
| TUG, s | | | | | | | | | | | |  |
| 6PBT (*n* = 14)^^^ | 13.0 ± 1.4 | 12.3 ± 1.4 | 11.8 ± 1.2 | 0.138 | 0.080 |  | <0.001 | 0.296 |  | 0.427 | 0.025 | |
| 2PBT (*n* = 13)^¶^ | 12.3 ± 1.5 | 9.9 ± 1.5 | 10.2 ± 1.3 |  |  |  |  |  |  |  |  | |
| SPPB, pt. | SPPB, pt. |  |  |  |  |  |  |  |  |  |  | |
| 6PBT (*n* = 14)^^^ | 9.1 ± 0.8 | 9.7 ± 0.7 | 9.5 ± 0.7 | 0.230 | 0.057 |  | 0.360 | 0.040 |  | 0.375 | 0.032 | |
| 2PBT (*n* = 13)^¶^ | 10.4 ± 0.8 | 10.3 ± 0.7 | 10.5 ± 0.8 |  |  |  |  |  |  |  |  | |
| *Physical activity* | | | | | | | | | | | |  |
| Mean daily energy expenditure, METs | | | | | | | | | | | |  |
| 6PBT (*n* = 8)^○^ | 1.37 ± 0.07 | 1.39 ± 0.08 | 1.41 ± 0.08 | 0.625 | 0.038 |  | 0.231 | 0.117 |  | 0.523 | 0.035 | |
| 2PBT (*n* = 6)^■^ | 1.29 ± 0.08 | 1.28 ± 0.10 | 1.39 ± 0.09 |  |  |  |  |  |  |  |  | |
| Mean daily duration in MVPA, min^†^ | | | | | | | | | | | |  |
| 6PBT (*n* = 8)^○^ | 201.4 ± 25.9 | 197.0 ± 21.8 | 228.1 ± 32.4 | 0.387 | 0.073 |  | 0.124 | 0.166 |  | 0.891 | 0.002 | |
| 2PBT (*n* = 6)^■^ | 173.2 ± 29.9 | 160.9 ± 25.1 | 178.3 ± 37.5 |  |  |  |  |  |  |  |  | |
| Mean daily step count^†^ | | | | | | | | | | | |  |
| 6PBT (*n* = 8)^○^ | 7416 ± 892 | 6871 ± 885 | 7314 ± 1151 | 0.912 | 0.008 |  | 0.382 | 0.077 |  | 0.735 | 0.010 | |
| 2PBT (*n* = 6)^■^ | 8187 ± 773 | 7909 ± 766 | 8452 ± 997 |  |  |  |  |  |  |  |  | |
| Maximum step count per walking bout | | | | | | | | | | | |  |
| 6PBT (*n* = 8)^○^ | 81 ± 10 | 90 ± 10 | 84 ± 10 | 0.053 | 0.217 |  | 0.520 | 0.053 |  | 0.709 | 0.012 | |
| 2PBT (*n* = 6)^■^ | 81 ± 11 | 70 ± 12 | 87 ± 11 |  |  |  |  |  |  |  |  | |
| *Concerns about falling* | | | | | | | | | | | |  |
| Short FES-I, pt. | | | | | | | | | | | |  |
| 6PBT (*n* = 14)^^^ | 9.6 ± 0.7 | 9.3 ± 0.6 | 9.4 ± 0.5 | 0.514 | 0.026 |  | 0.080 | 0.096 |  | 0.550 | 0.014 | |
| 2PBT (*n* = 13)^¶^ | 9.5 ± 0.7 | 8.7 ± 0.6 | 8.7 ± 0.5 |  |  |  |  |  |  |  |  | |
| *Executive functioning* | | | | | | | | | | | |  |
| TMT-A, s | | | | | | | | | | | |  |
| 6PBT (*n* = 14)^^^ | 74.9 ± 9.9 | 65.5 ± 7.6 | 62.4 ± 9.7 | 0.198 | 0.065 |  | 0.214 | 0.062 |  | 0.369 | 0.034 | |
| 2PBT (*n* = 12)^▼^ | 56.9 ± 10.7 | 52.1 ± 8.2 | 59.1 ± 10.5 |  |  |  |  |  |  |  |  | |
| TMT-B, s^†^ | | | | | | | | | | | |  |
| 6PBT (*n* = 14)^^^ | 171.7 ± 18.7 | 162.1 ± 22.3 | 134.8 ± 18.0 | 0.552 | 0.025 |  | 0.167 | 0.075 |  | 0.341 | 0.039 | |
| 2PBT (*n* = 12)^▼^ | 123.6 ± 21.1 | 140.4 ± 25.2 | 125.1 ± 20.3 |  |  |  |  |  |  |  |  | |
| Descriptive data given as estimated marginal means ± standard errors, with *p*-values calculated for repeated-measures ANOVA (within-subject factor = time [T1, T2, T3], between-subject factor = group [6PBT, 2PBT]). T1 = baseline assessment, T2 = post-intervention assessment, T3 = follow-up assessment, Brief-BESTest = Brief Balance Evaluation Systems Test, 6PBT = six-session perturbation-based balance training, 2PBT = two-session perturbation-based balance training and four-session conventional treadmill training, FSST = Four Square Step Test, 2MWT = 2-min walk test, TUG = Timed Up and Go, SPPB = Short Physical Performance Battery, Short FES-I = Short Falls Efficacy Scale-International, TMT = Trail Making Test.  ^†^Mean daily step count, mean daily MVPA duration, and TMT-B were analyzed after natural log-transformation due to non-normally distributed residuals.  ^‡^Five participants not included in the complete-case analysis due to dropout during the intervention period caused by unplanned eye surgery (*n* = 1) or acute health deterioration (*n* = 1), unwillingness to complete the T3 assessment (*n* = 2), and no Brief-BESTest at T1 due to exhaustion (*n* = 1).  ^#^Six participants not included in the complete-case analysis due to dropout after the baseline assessment caused by anxiety about receiving further perturbations during the study period (*n* = 4), dropout during the intervention period caused by an intervention-unrelated injurious fall (*n* = 1), and no Brief-BESTest at T1 due to exhaustion (*n* = 1).  ^§^Six participants not included in the complete case analysis due to dropout during the intervention period caused by unplanned eye surgery (*n* = 1) or acute health deterioration (*n* = 1), unwillingness to complete the T3 assessment (*n* = 2), no FSST at T2 due to exhaustion (*n* = 1), and no FSST at T2 and T3 due to physical limitations.  ^¶^Five participants not included in the complete-case analysis due to dropout after the baseline assessment caused by anxiety about receiving further perturbations during the study period (*n* = 4) or dropout during the intervention period caused by an intervention-unrelated injurious fall (*n* = 1).  ^$^Five participants not included in the complete-case analysis due to dropout during the intervention period caused by unplanned eye surgery (*n* = 1) or acute health deterioration (*n* = 1), unwillingness to complete the T3 assessment (*n* = 2), or technical issues with APDM at T1 (*n* = 1).  ^^^Four participants not included in the complete-case analysis due to dropout during the intervention period caused by unplanned eye surgery (*n* = 1) or acute health deterioration (*n* = 1), and unwillingness to complete the T3 assessment (*n* = 2).  ^○^Ten participants not included in the complete-case analysis due to dropout during the intervention period caused by unplanned eye surgery (*n* = 1) or acute health deterioration (*n* = 1), unwillingness to complete the T3 assessment (*n* = 2), invalid sensor wear time at T1 (<600 min) (*n* = 1), and refusal of PA monitoring (*n* = 5).  ^■^Twelve participants not included in the complete-case analysis due to dropout after the baseline assessment caused by anxiety about receiving further perturbations during the study period (*n* = 4), dropout during the intervention period caused by an intervention-unrelated injurious fall (*n* = 1), and refusal of PA monitoring (*n* = 7).  ^▼^Five participants not included in the complete-case analysis due to dropout after the baseline assessment caused by anxiety about receiving further perturbations during the study period (*n* = 4), dropout during the intervention period caused by an intervention-unrelated injurious fall (*n* = 1), and unwillingness to complete TMT B at T2. | | | | | | | | | | | |  |

**Secondary outcomes**

The complete case analysis revealed no significant interaction effects on global and dynamic balance, gait capacity, functional mobility, physical activity, concerns about falling, and executive functioning (*p*=0.053-0.912). However, significant moderate to large time effects were found for dynamic balance (FSST), gait capacity (gait speed, cadence, step time, stride length, double support), and functional mobility (TUG) (*p*<0.004-0.034; *η_p_^2^*=0.138-0.296).

Post-hoc tests for multiple comparisons revealed no significant differences between assessment time points for FSST (T1 vs. T2: *p_Bonf_*=0.180, T1 vs. T3: *p_Bonf_=*0.084; T2 vs. T3: *p_Bonf_=*0.379), cadence (T1 vs. T2: *p_Bonf_=*0.093, T1 vs. T3: *p_Bonf_=*0.258; T2 vs. T3: *p_Bonf_=*0.535), and step time (T1 vs. T2: *p_Bonf_=*0.079, T1 vs. T3: *p_Bonf_=*0.354; T2 vs. T3: *p_Bonf_=*0.659). In contrast, stride length (T1 vs. T2: *p_Bonf_*=0.022; T1 vs. T3: *p_Bonf_=*0.012) and TUG (T1 vs. T2: *p_Bonf_=*0.004; T1 vs. T3: *p_Bonf_=*0.006) showed significant improvements over the intervention period, which were maintained at follow-up. Gait speed improved significantly from T1 to T2 *p_Bonf_* *p=*0.026), but this improvement was not maintained at T3 (*p_Bonf_=*0.052). Double support showed significant improvement from T1 to T3 (*p_Bonf_=*0.019), but no significant change from T1 to T3 (*p_Bonf_*=0.233).

**Acceptability**

The complete case analysis (6PBT: *n*=16; 2PBT: *n*=13) revealed a similarly high acceptability of the PBT in both groups, with mean TFA questionnaire scores in the upper quartiles (6PBT= 26.8±4.2 pt., 2PBT = 28.0±5.7 pt.) and no significant between-group difference (*p*=0.428).
